# Allo-parapatric speciation goes offshore

**DOI:** 10.1093/nsr/nwy110

**Published:** 2018-10-15

**Authors:** Trevor Price

**Affiliations:** 1 Department of Ecology and Evolution, University of Chicago, USA; 2 Recommender of NSR

A basic conundrum in speciation is that isolation is required for differentiation, but dispersal is required to produce populations in different places, which can then diverge [1]. These conflicting pressures seem to make the optimal conditions for speciation to be those in which barriers appear and disappear on the right timescale. If barriers break down too quickly, divergence will be incomplete and populations will merge; if they appear too slowly, few species will form. The timescale of barrier fluctuations is known in some cases, such as from sea-level fluctuations. In this novel application of genomics and computer modeling, He *et al.* connect paleoclimates to recent mangrove speciation and differentiation. They demonstrate that speciation has occurred despite periods of contact. By showing that genetic differentiation along the genome is too variable to be accounted for by a simple allopatric model, they confirm that gene flow between the diverging species must have happened, with timescales for contact on the order of 100 000  years. Further, they use a novel approach to estimate the minimum time for complete gene flow to be cut off if divergence were to happen entirely in allopatry, through comparisons of subspecies, and show that this is too long to be a feasible means of creating species diversity.

The paper combines cutting-edge genomic analysis with an impressive dataset to draw strong conclusions about times needed to create enough divergence that populations will not collapse back into one upon coming into contact. It also leads to increased understanding of mechanisms producing the extraordinary marine diversity in the region. Finally, the results set the stage for asking whether contact between divergent forms, such as in hybrid zones, could actually accelerate speciation rather than retard it, such as by promoting chromosomal inversions [2] or driving pre-zygotic reinforcement once some post-zygotic incompatibilities have developed [3].
